# Ground reaction forces during double limb stances while walking in individuals with unilateral transfemoral amputation

**DOI:** 10.3389/fbioe.2022.1041060

**Published:** 2023-01-12

**Authors:** Toshiki Kobayashi, Mark W. P. Koh, Abu Jor, Genki Hisano, Hiroto Murata, Daisuke Ichimura, Hiroaki Hobara

**Affiliations:** ^1^ Department of Biomedical Engineering, Faculty of Engineering, The Hong Kong Polytechnic University, Hong Kong, China; ^2^ Department of Leather Engineering, Faculty of Mechanical Engineering, Khulna University of Engineering & Technology, Khulna, Bangladesh; ^3^ Department of Systems and Control Engineering, Tokyo Institute of Technology, Tokyo, Japan; ^4^ Research Fellow of Japan Society for the Promotion of Science (JSPS), Tokyo, Japan; ^5^ Artificial Intelligence Research Center, National Institute of Advanced Industrial Science and Technology, Tokyo, Japan; ^6^ Department of Mechanical Engineering, Tokyo University of Science, Chiba, Japan; ^7^ Faculty of Advanced Engineering, Tokyo University of Science, Tokyo, Japan

**Keywords:** above-knee prosthesis, amputee, walking, gait, GRF, kinetics

## Abstract

The asymmetrical gait of individuals with unilateral transfemoral amputation has been well documented. However, there is not a wealth of investigation into asymmetries during the double limb stance depending on whether the intact or prosthetic limb is leading. The first aim of this study was to compare ground reaction forces during the double limb stance of individuals with unilateral transfemoral amputation depending on whether their intact (initial double limb stance) or prosthetic (terminal double limb stance) limb was leading. The second aim of this study was to compare the asymmetry ratio of ground reaction forces during the double limb stance between individuals with and without unilateral transfemoral amputation. Thirty individuals, fifteen with unilateral transfemoral amputation and fifteen who were able-bodied, were recruited for this study. Each individual walked on an instrumented treadmill for 30 s at eight different speeds, ranging from 2.0 km/h to 5.5 km/h with .5 km/h increments. Ground reaction force parameters, temporal parameters, and asymmetry ratios of all parameters were computed from the data collected. The appropriate statistical analyses of all data based on normality were conducted to investigate the aims of this study. Significant main effects of speed, double limb stance, and their interactions were found for most parameters (*p* < .01 or *p* < .05). Individuals with unilateral transfemoral amputation spent a longer duration in terminal double limb stance than initial double limb stance at all tested speeds. They also experienced significantly higher peak vertical ground reaction force during initial double limb stance compared to terminal double limb stance with increasing walking speed. However, during terminal double limb stance, higher anteroposterior ground reaction force at initial contact was found when compared to initial double limb stance. Significant differences between individuals with unilateral transfemoral amputation and able-bodied individuals were found in asymmetry ratios for peak vertical ground reaction force, anteroposterior ground reaction force, anteroposterior shear, and mediolateral shear at all tested speeds. Asymmetrical loading persists in individuals with unilateral transfemoral amputation during double limb stance. Increasing walking speed increased ground reaction force loading asymmetries, which may make individuals with unilateral transfemoral amputation more susceptible to knee osteoarthritis or other musculoskeletal disorders. Further study is necessary to develop ideal gait strategies for the minimization of gait asymmetry in individuals with unilateral transfemoral amputation.

## 1 Introduction

The human gait cycle has been generally accepted to consist of two periods known as stance and swing, and the portion of the stance period where both limbs are in contact with the ground has been defined as the double limb stance (DLS) (Perry, 2010). However, the forces generated by each limb to support the body weight during the time spent in DLS vary unequally. Studies have shown that even in able-bodied individuals, ground reaction force (GRF) asymmetries have been observed between the lower limbs during gait (Herzog et al., 1989; Sadeghi et al., 1997). For propulsion in walking, the center of mass (CoM) displacement could be affected by the forces produced by the leading and trailing limbs during the DLS (Donelan et al., 2002; Soo and Donelan, 2012). The adaptation of the two limbs by the central nervous system to the factors required to manage oscillating gait, such as gravity and CoM height, in order to control the movement of the CoM during walking (Brenière, 1996), could result in GRF asymmetries.

The biomechanical characteristics of gait between individuals with and without unilateral transfemoral amputation (uTFA) have noticeable differences. Since individuals with uTFA have a prosthetic limb, the differences in GRFs generated by the lower limbs are likely exacerbated, leading to asymmetrical gait. The asymmetrical gait of individuals with uTFA has been well documented (Burkett et al., 2003; Castro et al., 2014; Soares et al., 2016; Rutkowska-Kucharska et al., 2018), and has been correlated to health conditions that can be developed such as osteoarthritis (Nolan et al., 2003; Welke et al., 2019) and various musculoskeletal degenerative disorders (Kulkarni et al., 1998; Norvell et al., 2005; Devan et al., 2014). In individuals with uTFA, there have been noted kinematic inequalities between the two DLS, depending on whether the intact or prosthetic limb is leading (Jaegers et al., 1995). Therefore, the GRFs generated by each limb during DLS are also likely to have inequalities in individuals with uTFA. This is most likely due to the asymmetrical gait as well as the anatomical and functional differences between the intact and prosthetic limbs in individuals with uTFA.

Although DLS only accounts for 20%–25% of the gait cycle, it is valuable for the assessment of gait and diagnosis of disease (Davis and Cavanagh, 1993; Goldberg et al., 2006). During DLS, there are robust responses to perturbations, indicating active control (Vlutters et al., 2016; Reimann et al., 2018; Vlutters et al., 2018). Incorrect shifting of body mass which occurs during DLS is the most common cause of falls (Robinovitch et al., 2013). And the DLS is reported to be associated with improved stability of gait due to the better control of CoM movement (Williams and Martin, 2019). The displacement of CoM could be initiated by the leading or trailing limbs during the DLS, as larger anteroposterior (AP) to vertical GRF (vGRF) ratios were found when compared to the single stance phase (Vielemeyer et al., 2021) to possibly commence forward movement. This is further supported as peaks can be found for the sum of GRFs during DLS indicating a higher acceleration of CoM during this time period (Uchida and Delp, 2021).

The total GRF is mostly composed of the vertical component during the DLS, with small anteroposterior components at the initial contact (IC) and toe-off of the DLS respectively (Uchida and Delp, 2021). As walking intends to propel the CoM in the forward direction, the anteroposterior components of forces also should likely contribute (Sato and Yamada, 2017). Although escalating walking speed increases vGRF peaks measured during the DLS, which affect upward and downward acceleration of CoM, in able-bodied individuals (Kirtley, 2006), no significant effects in anteroposterior components were found (Sato and Yamada, 2018). Moreover, in individuals with uTFA, mediolateral (ML) components of GRFs during single-limb stance are significantly associated with gait symmetry (Hisano et al., 2021). During the DLS, the GRFs produced in individuals with uTFA are the sum of the intact limb GRF and the prosthetic limb GRF. As the position of the prosthetic limb and intact limb switch between the two DLS, there are likely differences in the forces generated by the different limbs during the two DLS. Therefore, there is a pertinent need to understand the strategies by which GRFs under both limbs are coordinated during DLS and how individuals with uTFA employ such strategies to stabilize body movements.

The asymmetrical gait of individuals with uTFA has been well documented, with a number of studies documenting parameters measured during the DLS. However, studies that had investigations during the DLS focused mostly on time variables. A study conducted on individuals with transtibial amputation (Isakov et al., 1997) found that there was a significantly longer time spent in the DLS when the prosthetic limb was leading. For individuals with uTFA, the amount of time spent in the DLS decreases with increasing walking speed, similar to able-bodied individuals (Schaarschmidt et al., 2012). But, as walking speed increases, the time spent in the DLS becomes significantly shorter while the prosthetic limb is leading compared to the intact limb leading or in able-bodied individuals (Amma et al., 2021). Hence, GRF variables should be altered with increasing walking speed due to the shortened time of the DLS. However, it is still unclear how GRFs alter during the DLS with increasing speed, particularly in those with uTFA. But, to the best of our knowledge, no study to date has analyzed GRFs components in detail during DLS across a range of walking speeds in individuals with uTFA. Thus, studying GRFs during the DLS could be a possible key toward understanding the adaptation and compensation strategies of gait in individuals with uTFA.

Therefore, the present study investigates GRFs incorporating vertical, anteroposterior, and mediolateral components during the two DLS. The effect of changing gait speed on GRFs will also be analyzed to explore the fundamental function of the DLS in the adaptation and compensation of gait in individuals with uTFA. The initial DLS has been defined as when the intact limb is leading, and the terminal DLS has been defined as when the prosthetic limb is leading. The specific aims of this study were 1) to compare GRFs between the initial and terminal DLS in individuals with uTFA, and 2) to compare the asymmetry ratio of GRFs between individuals with uTFA and those without amputation in order to clarify their adaptation and compensation strategies of gait. This study’s first hypothesis is that the vertical, anteroposterior, and mediolateral components of GRF between the initial and terminal DLS will differ in individuals with uTFA because the leading limb switches between the intact and prosthetic limb. We expected that the vertical GRF would be larger at the initial DLS because the intact limb would allow more loading in early stance as the leading limb, while the anterior-posterior GRF would be larger at the terminal DLS because the intact limb would allow more push-off force in late stance as the trailing limb. The second hypothesis is that the magnitude of the asymmetry ratio of GRFs would be greater in individuals with uTFA when compared to able-bodied individuals without amputation.

## 2 Materials and methods

### 2.1 Participants

Thirty individuals consisting of fifteen individuals with uTFA and fifteen without amputation to form a control group were recruited for this study (Table 1). The following criteria were applied to recruit individuals with uTFA: 1) absence of neuromuscular disorders, 2) no significant functional limitations in either lower-limb, 3) lightly active or higher, and 4) classified at K3 or K4 functional level (Borrenpohl et al., 2016). The participants for the control group were recruited to match the general characteristics of the individuals with uTFA group such as the age (uTFA group = 30 ± 8 years, control group = 30 ± 9 years), gender (11 males and four females for both groups), body mass (uTFA group = 66.7 ± 15.7 kg, control group = 68.7 ± 10.1 kg), and height (uTFA group = 1.65 ± .09 m, control group = 1.67 ± .07 m). The body mass of the individuals with uTFA includes the mass of their prosthesis. All study procedures conducted followed the guidelines outlined by the Declaration of Helsinki (1983) and were approved by the local ethics committee. The contents of the study were made known to the participants, and their consent was acquired.

**TABLE 1:** Demographic characteristics.

Participant	Sex	Age (years)	Height m)	Mass (kg)	BMI	Time since amputation (years)	Prosthetic knee unit	Prosthetic feet	Amputated limb	Cause of amputation
Individual with UTFA										
1	F	27	1.54	46.4	19.5	1	3R60	RS2000 Runway	Right	Trauma
2	M	23	1.68	58.3	20.7	20	3R80	Vari-Flex	Left	Cancer
3	M	43	1.67	57.6	20.7	8	3R106	Triton	Left	Cancer
4	F	21	1.49	47.5	21.4	10	3R106	Total Concept	Right	Sarcoma
5	M	31	1.72	64.0	21.6	8	Mauch knee	Elation	Right	Sarcoma
6	F	21	1.52	52.1	22.5	13	3R106	Elation	Left	Sarcoma
7	M	27	1.75	71.0	23.2	6	3R80	1C64 Triton	Right	Trauma
8	M	36	1.61	60.1	23.2	18	3R106	Triton	Right	Trauma
9	F	20	1.56	56.4	23.2	6	Total knee	Vari-Flex XC	Right	Trauma
10	M	34	1.61	61.4	23.7	21	3R95	Vari-Flex	Left	Sarcoma
11	M	30	1.70	70.3	24.3	21	3R106	Vari-Flex	Right	Sarcoma
12	M	29	1.65	70.1	25.7	8	3R80	1C61 Triton	Right	Trauma
13	M	42	1.70	75.4	26.1	32	3R80	Highlander	Right	Trauma
14	M	17	1.77	84.0	26.8	3	NK-6	Triton	Right	Congenital
15	M	42	1.75	111.1	36.3	25	3R80	Dyna Trek	Right	Trauma
Mean		30	1.65	65.7	23.9	13				
SD		8	.09	15.7	3.9	9				
Control										
1	F	28	1.63	52.9	19.9					
2	F	23	1.59	54.2	21.5					
3	M	22	1.70	63.1	21.8					
4	M	28	1.78	72.1	22.8					
5	M	51	1.67	65.3	23.4					
6	F	21	1.57	58.3	23.6					
7	M	21	1.66	65.6	23.8					
8	M	49	1.76	74.6	24.1					
9	F	23	1.61	62.5	24.1					
10	M	26	1.72	71.6	24.2					
11	M	25	1.60	61.9	24.2					
12	M	32	1.71	80.5	27.5					
13	M	38	1.70	79.6	27.5					
14	M	31	1.77	88.6	28.3					
15	M	34	1.62	79.4	30.3					
Mean		30	1.67	68.7	24.5					
SD		9	.07	10.1	2.7					

### 2.2 Task and procedure

This study was carried out on a split-belt force-instrumented treadmill (FTMH-1244WA, Tec Gihan, Kyoto, Japan). The treadmill was equipped with a safety harness to alleviate participants’ fears of falling. In addition, the safety harness was adjusted to provide sufficient slack to prevent any impact on each participant’s normal walking gait. For the test, participants were asked to begin walking for 30 s at each of eight different speeds, ranging from 2.0 km/h to 5.5 km/h with .5 km/h increments. Before starting the test, each participant familiarized themselves with testing conditions by rehearsing walking on the treadmill for 7 min at a minimum (Zeni and Higginson, 2010; Meyer et al., 2019). During this familiarization period, all participants experienced each of the eight walking speeds until they could walk comfortably at each speed. With real-time data collection and video recordings, each participant was confirmed to be able to walk confidently at all eight different speeds without the aid of the handrails on the treadmill. In between trials, participants were allowed rest periods as requested to diminish the effects of fatigue.

### 2.3 Data collections and analyses

**FIGURE 1:**
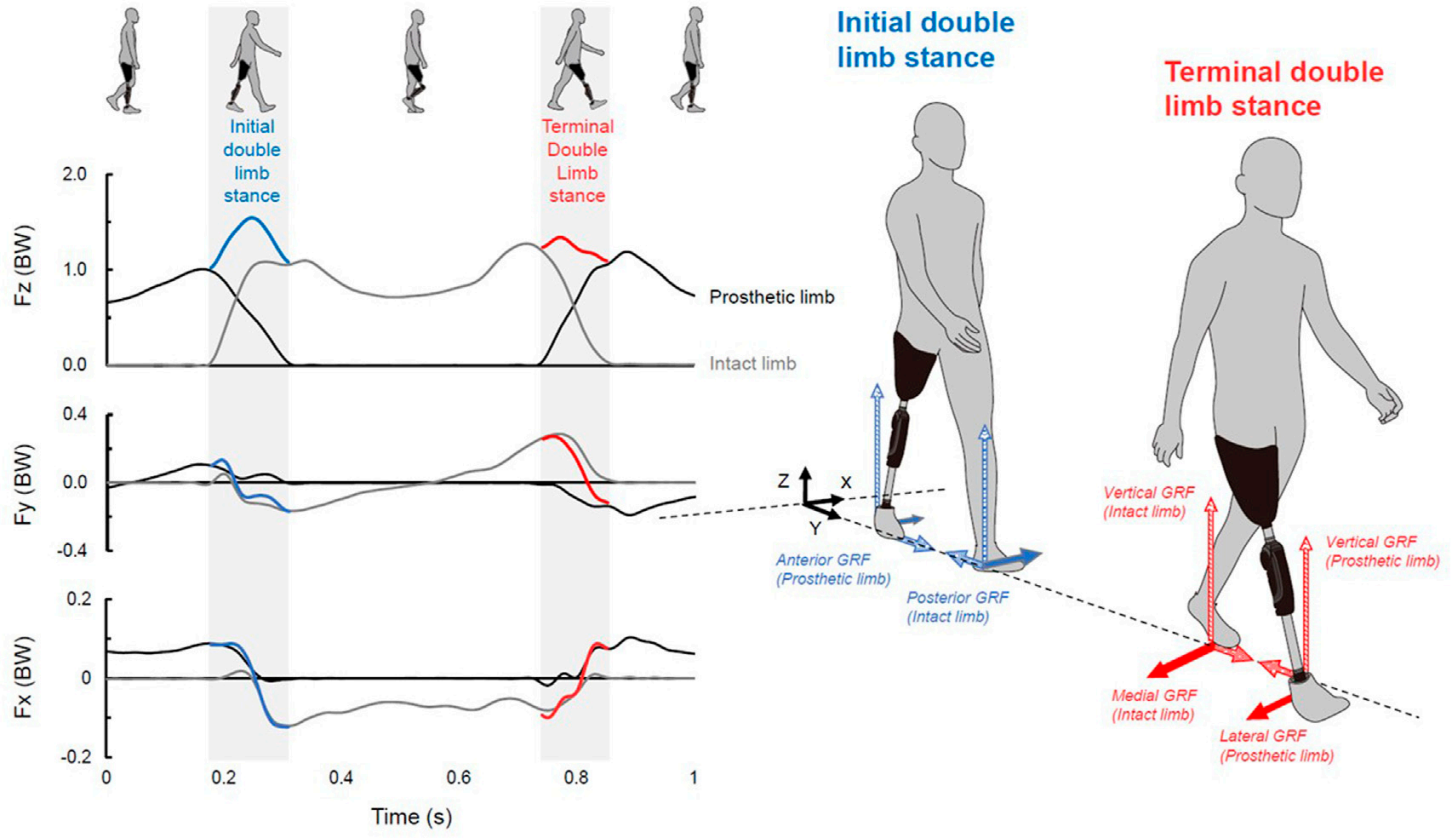
Left: vertical (Fz), anteroposterior (Fy), and mediolateral (Fx) ground reaction forces (GRFs) from the perspective of the leading limb normalized to the body weight (BW). GRFs were recorded from one representative trial of an individuals with uTFA during walking at 4.5 km/h. Bold blue and red curves indicate the sum of the GRF of the initial and terminal double limb stance in each component, respectively. Right: definition of each GRF component at initial and terminal double limb stance.

Two piezoelectric force plates (TF-40120-CL and TF-40120-CR, Tec Gihan, Kyoto, Japan) with six-degrees-of-freedom were implanted in the treadmill to acquire GRF values (vertical: Fz, anteroposterior or AP: Fy, mediolateral or ML: Fx) at a sampling frequency of 1,000 Hz (Figure 1). The heel strike (or initial contact) and toe-off of the stance phase during walking were determined by setting 40 N of the vGRF as a threshold value. For each participant, around 27 ± 7 consecutive steps at each walking speed were recorded to calculate the dependent variables at the initial and terminal DLS (Figure 1) and the average of the parameters over the participants was calculated. The initial DLS has been defined as the intact limb leading for those with uTFA, while the terminal DLS has been defined as the prosthetic limb leading (Figure 1). The initial DLS and terminal DLS for individuals without amputation have been defined as the left limb leading and the right leg leading, respectively. In individuals with uTFA and the control group, GRFs parameters during the initial and terminal DLS were analyzed. The GRF data were filtered using a fourth-order low-pass zero-lag Butterworth filter at a cut-off frequency of 20 Hz and normalized by the participants’ body mass (Hoffman and Donaghe, 2011).

Data from the GRF parameters (peak vGRF, AP GRF at IC, and ML GRF at IC) and temporal parameters (DLS time, % DLS of peak vGRF timing, % DLS of AP shear, and % DLS of ML shear) during the initial and terminal DLS were extracted and analyzed. For ML GRF at IC, because lateral direction is opposite for each limb, absolute value was used. % DLS from 0% (leading limb IC) to 100% (trailing limb toe-off) was used to normalize the time spent during the DLS, enabling the analysis of temporal parameters. The % DLS of AP and ML shear was defined as the % of DLS when the AP and ML GRF registered switched between anterior to posterior and lateral to medial, respectively. ML directions were determined from the perspective of the leading limb. Subsequently, the asymmetry ratio of each parameter was calculated for the individuals with uTFA and the control group (Silverman et al., 2008). The asymmetry ratio was calculated as the value of each parameter at the terminal DLS divided by the value of each parameter at the initial DLS. Therefore, a greater asymmetry between the initial and terminal DLS is indicated by a smaller or larger asymmetry ratio than 1.

### 2.4 Statistical analysis

All of the data was analyzed using SPSS statistical tool (IBM SPSS Statistics Version 26, IBM, Armonk, NY, United States). Data normality for each gait parameter and asymmetry ratio was confirmed by the Shapiro-Wilk test. A two-way mixed analysis of variance (ANOVA) was performed for each gait parameter that demonstrated a normal distribution (within-subject: speed; between-subject: initial DLS and terminal DLS), and a Bonferroni test was employed as a post-hoc comparison. For an asymmetry ratio with a normal distribution (within-subject: speed, between-subject: uTFA and control), a two-way mixed ANOVA and a Bonferroni post-hoc comparison were also carried out. Adversely, the Wilcoxon signed ranked tests and Friedman tests were used to investigate the primary effects of the DLS and speeds for gait parameters and asymmetry ratios that were not normally distributed. To establish significance, post-hoc comparisons considering the DLS effects were conducted using the Mann-Whitney U test, and the Wilcoxon signed-rank test was used for speed effects. Statistical significance was defined as *p* < .05 in all statistical tests used in this study.

## 3 Results

### 3.1 Main effects of the speed, DLS, and their interactions

There were significant speed effects for most parameters (*p* < .01 or *p* < .05) except for ML GRF at IC (*p* = .760) and peak vGRF timing asymmetry (*p* = .698) (Table 2). There were significant DLS effects for most of the valid parameters (*p* < .01) except for peak vGRF timing (*p* = .500). All parameters displayed interaction effects of speed and DLS (*p* < .01) except for peak vGRF asymmetry (*p* = .050).

**TABLE 2:** Main effects of speed and DLS by statistical tests for gait parameters and asymmetry ratios.

	Main effect of speed	Main effect of DLS	Interaction	Normality
Peak vGRF asymmetry	*p* < .01	*p* < .01	*p* = .050	Y
AP GRF at IC	*p* < .01	*p* < .01	*p* < .01	Y
Peak vGRF timing (%DLS)	*p* < .01	*p* = .500	*p* < .01	Y
AP shear (%DLS)	*p* < .01	*p* < .01	*p* < .01	Y
ML shear (%DLS)	*p* < .01	*p* < .01	*p* < .01	Y
DLS (s)	*p* < .01	—	—	N
DLS asymmetry	*p* < .05	—	—	N
Peak vGRF (BW)	*p* < .01	—	—	N
AP GRF at IC asymmetry	*p* < .01	—	—	N
ML GRF at IC	*p* = .760	—	—	N
ML GRF at IC asymmetry	*p* < .01	—	—	N
Peak vGRF timing asymmetry	*p* = .698	—	—	N
AP shear asymmetry	*p* < .01	—	—	N
ML shear asymmetry	*p* < .01	—	—	N

Y represented “yes”: a parametric test was used to test this parameter. N represented “no”: a non-parametric test was used to test this parameter. Abbreviations: DLS, double limb stance; IC, initial contact.

### 3.2 Effects of walking speeds on GRFs during initial and terminal DLS

**FIGURE 2:**
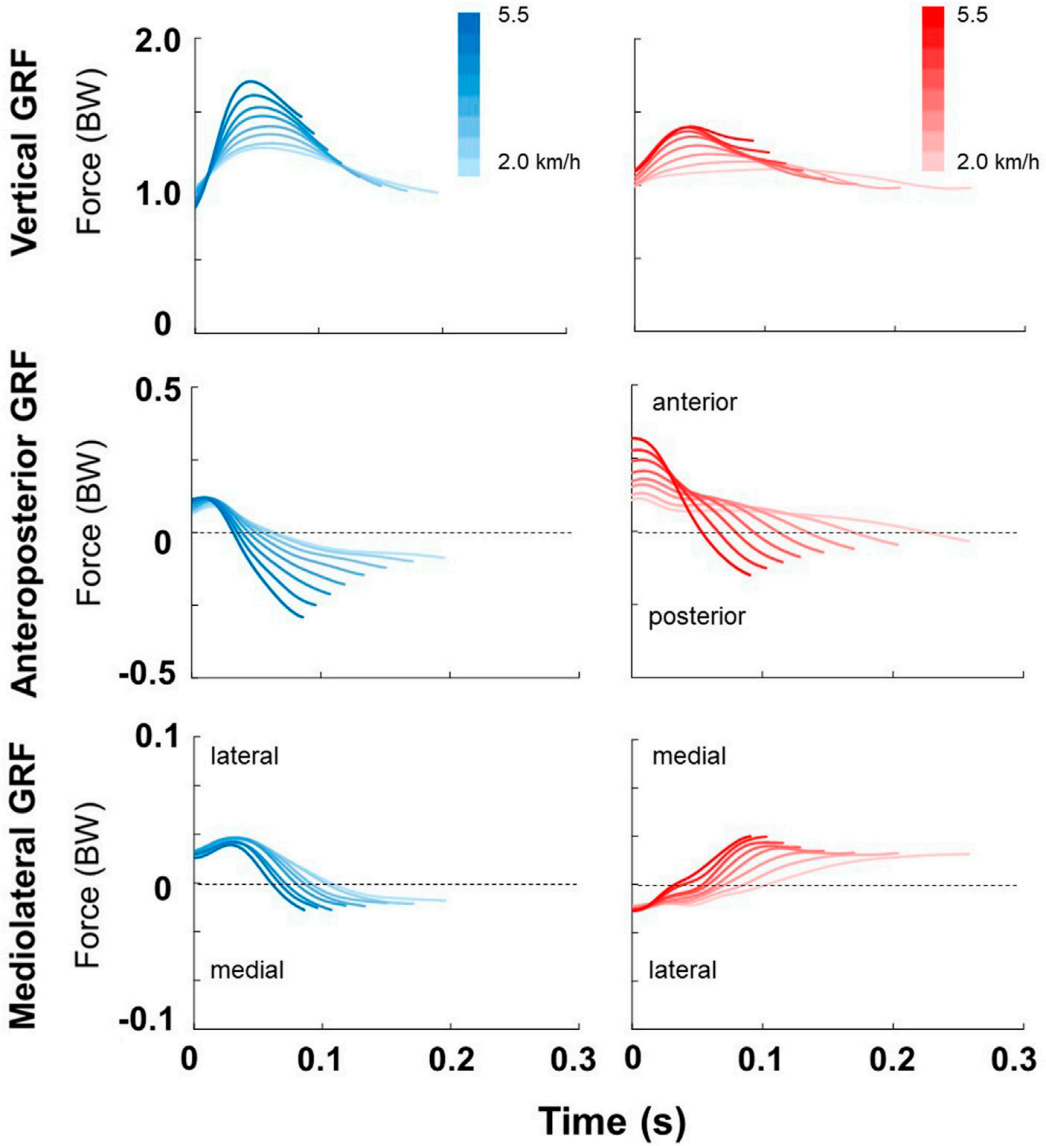
From top to bottom; vertical, anteroposterior, and mediolateral GRF of both the initial (blue) and the terminal (red) double limb stance across a range of walking speeds, respectively. Mediolateral directions are from the perspective of the leading limb during the DLS. The data was the average of 15 individuals with uTFA. In each plot, the vertical axis is normalized to the subject’s body weight (BW). The darkest and lightest colors represent the fastest (5.5 km/h) and slowest (2.0 km/h) walking speeds, respectively.

The peak vGRF during the initial DLS is higher than the terminal DLS at all speeds (Figure 2). As the walking speed increases, the peak vGRF increases in response. Regarding anteroposterior GRF, during the initial DLS, the peak anterior GRF remains relatively constant, while at increasing speeds, the terminal anterior GRF increases. The peaks of anterior GRF are generally higher in terminal DLS when compared to initial limb stance, but the posterior GRF peaks were higher during the initial DLS at all walking speeds. The time transitioning from anterior to posterior GRF (i.e., AP shear) was also shorter during the initial DLS when compared to the terminal DLS at all speeds. The transition in mediolateral GRF (i.e., ML shear) generally occurred at similar periods of time when comparing the initial DLS to the terminal DLS. The peak lateral GRF during the initial DLS is higher than that of the terminal DLS at all walking speeds. However, the peak medial GRF respective to the leading limb is higher during terminal DLS at all walking speeds.

### 3.3 Effects of walking speeds on initial and terminal DLS duration

**FIGURE 3:**
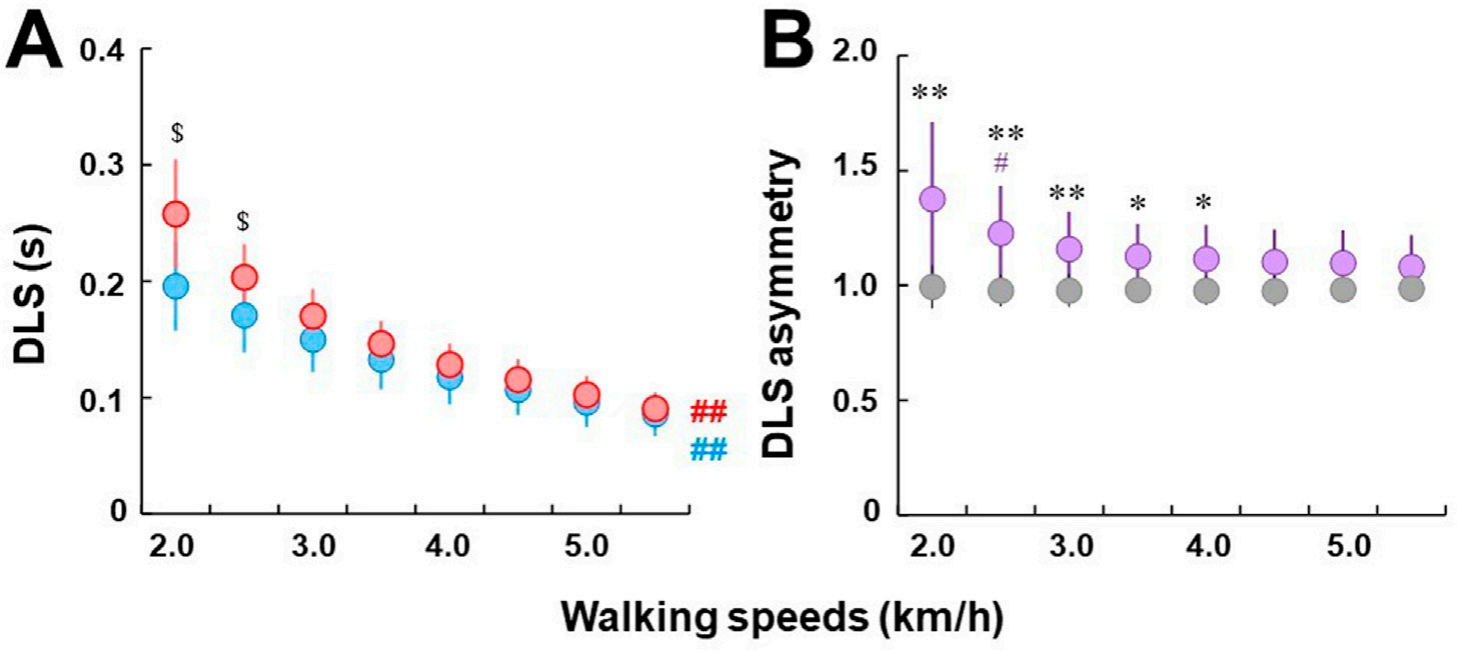
**(A)** Comparisons of double limb stance across a range of speeds. Blue and red circles indicate initial and terminal double limb stance, respectively. Circles and error bars indicate the mean value and the standard deviation in each DLS, respectively. $ represents a significant difference between initial and terminal double limb stance at *p* < .05. Bold ## indicates significant differences from adjacent speeds at all tested speeds at *p* < .01. **(B)** Comparisons of double limb stance asymmetry between individuals with uTFA (purple circles) and control group (gray circles). Circles and error bars indicate the mean value and the standard deviation in each group, respectively. # represents a significant difference between the current and previous speeds at *p* < .05. * and ** represent significant differences in asymmetry ratio between the individuals with UTFA and control groups at *p* < .05 and .01, respectively.

The duration of terminal DLS was higher than the initial DLS for all walking speeds for individuals with uTFA with significant differences for walking speeds at 2.5 km/h or less (*p* < .05) (Figure 3A). A significant difference in the duration spent in the initial or terminal DLS was found from adjacent speeds at all walking speeds (*p* < .01).

Individuals with uTFA showed clear DLS asymmetry for all walking speeds, whereas in the control group, it was almost constant at 1.0 with minimal to no DLS asymmetry ratio (Figure 3B). However, DLS asymmetry in individuals with uTFA was decreased in response to increasing walking speeds. When compared to the control group, individuals with uTFA displayed significant differences in DLS asymmetry at 4.0 km/h or less (*p* < .01 or *p* < .05). Significant differences between previous and current speeds were only found at 2.5 km/h for DLS asymmetry in individuals with uTFA (*p* < .05).

### 3.4 Effects of walking speeds on GRF magnitude parameters during initial and terminal DLS

**FIGURE 4:**
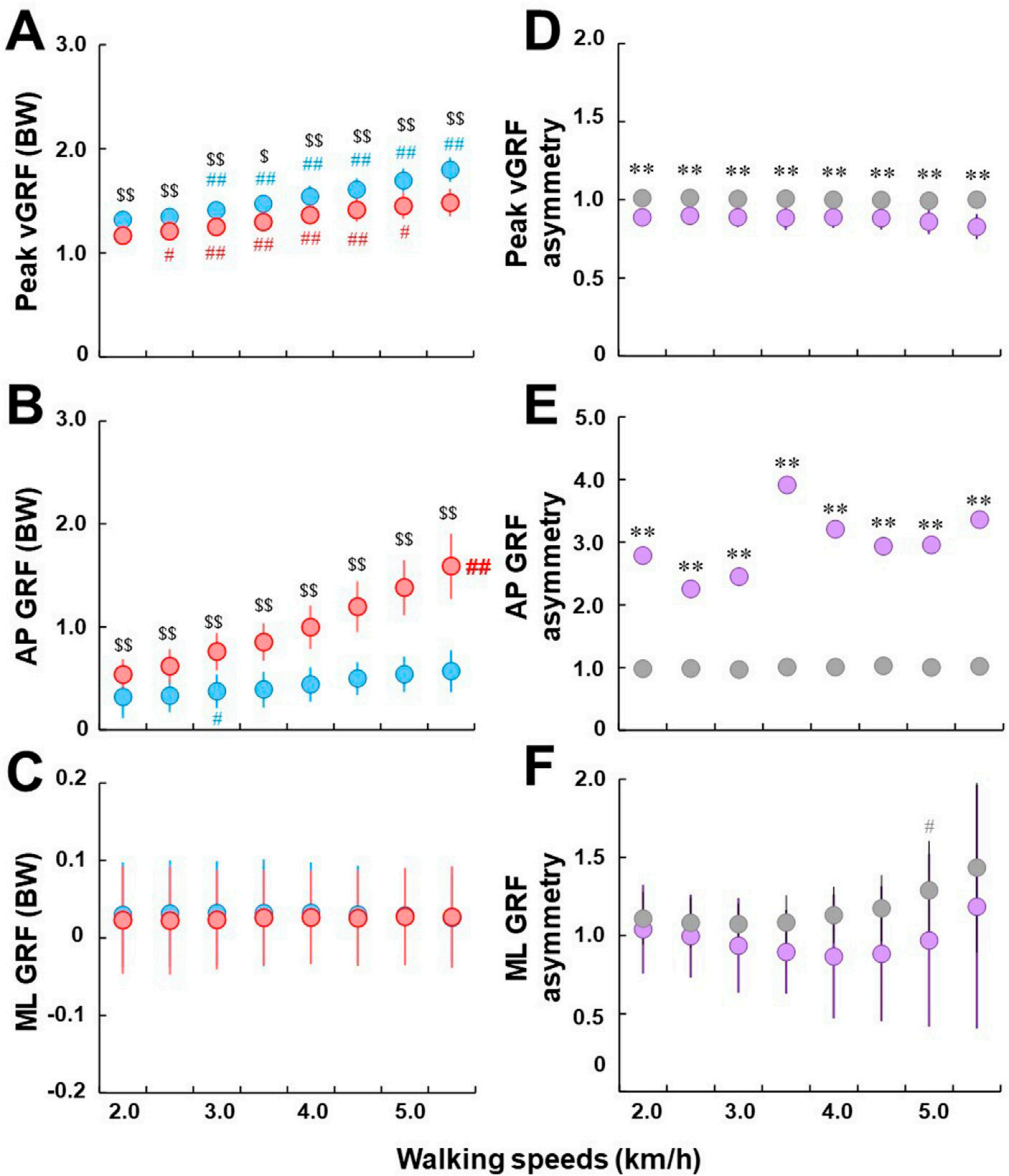
**(A–C)**: Comparisons of peak vGRF **(A)**, anteroposterior GRF **(B)**, and mediolateral GRF **(C)** between initial (blue) and terminal double limb stance (red), respectively. All GRF parameters were normalized to each participant’s body weight (BW). Circles and error bars indicate the mean value and the standard deviation in each DLS, respectively. For ML GRF, because lateral direction is opposite for each limb, absolute value was used. # and ## represent significant differences between the current and previous speeds at *p* < .05 and *p* < .01, respectively. $ and $$ represent significant differences between initial and terminal double limb stance at *p* < .05 and *p* < .01, respectively. Bold ## indicates a significant difference from adjacent speeds at all tested speeds at *p* < .01. **(D–F)**: comparisons of peak vGRF asymmetry **(D)**, AP GRF asymmetry **(E)**, and ML GRF asymmetry **(F)** between individuals with uTFA (purple) and the control group (gray). Circles and error bars indicate the mean value and the standard deviation in each group, respectively. For the plot **(E)**, standard deviations for the individuals with uTFA were omitted from the plot due to their large magnitude. * and ** represent significant differences in asymmetry ratio between the individuals with UTFA and control groups at *p* < .05 and .01, respectively.

For individuals with uTFA, significantly higher peaks of vGRF (peak vGRF) were observed during the initial DLS compared with the terminal DLS at all walking speeds (*p* < .01 or *p* < .05) (Figure 4A). As walking speed increased for individuals with uTFA, the peak vGRF observed for both the initial and terminal DLS increased in response. There were significant differences between current and previous speeds of peak vGRF at all tested walking speeds at 3.0 km/h or above for the initial DLS, and from 2.5 km/h to 5.0 km/h for the terminal DLS (*p* < .01 or *p* < .05).

Anteroposterior GRF (AP GRF at IC) was significantly higher during the terminal DLS in response to increased walking speeds when compared to the initial DLS (*p* < .01) (Figure 4B). However, only for the terminal DLS were there significant differences from adjacent speeds at all tested speeds (*p* < .01). For the initial DLS, only at 3.0 km/h were there significant differences between current and previous speeds observed (*p* < .05).

There were no significant differences observed in mediolateral GRF (ML GRF at IC) between the initial and terminal DLS (Figure 4C). There were also no significant differences observed between adjacent speeds at all tested walking speeds for both the initial and terminal DLS in individuals with uTFA.

There were significant differences at all walking speeds between individuals with uTFA and the control group of peak vGRF asymmetry (*p* < .01) (Figure 4D). While the control group stayed close to 1.0 at all walking speeds, the peak vGRF asymmetry ratio of individuals with uTFA was below 1.0.

There were significant differences at all walking speeds between individuals with uTFA and the control group of AP GRF asymmetry (*p* < .01) (Figure 4E). The control group AP GRF asymmetry stayed close to 1.0, with the AP GRF asymmetry of individuals with uTFA ranging between roughly 2.0 to 4.0. It should be noted that standard deviations for the individuals with uTFA were omitted from the plot due to their large magnitude.

For ML GRF asymmetry, there was no significant difference observed between individuals with uTFA and the control group at all tested walking speeds (Figure 4F). At 5.0 km/h in the control group, there was a significant difference between current and previous walking speeds in the ML GRF asymmetry ratio (*p* < .05).

### 3.5 Effects of walking speeds on GRF temporal parameters during initial and terminal DLS

**FIGURE 5:**
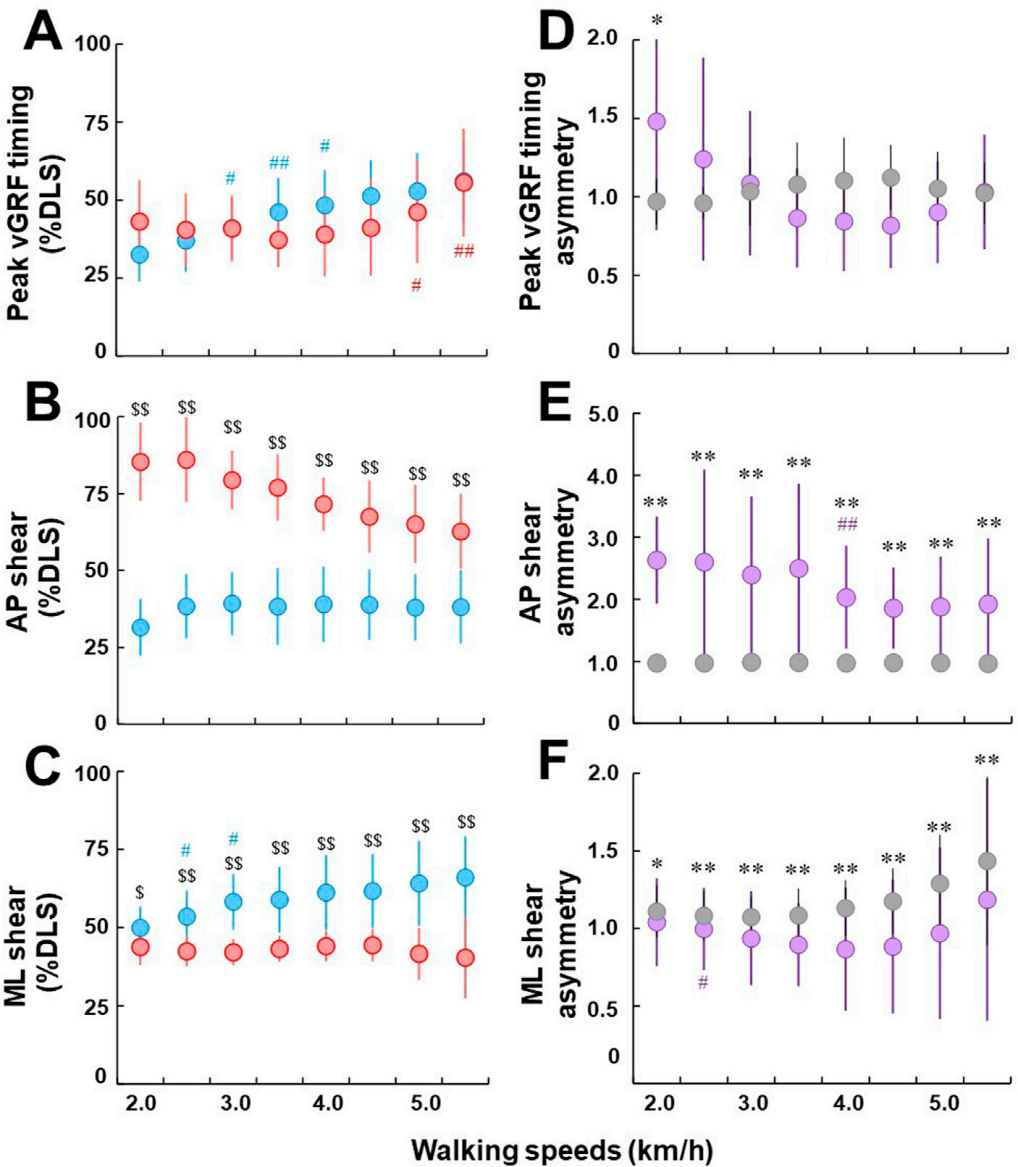
**(A–C)**: Comparisons of peak vGRF timing **(A)**, AP shear **(B)**, and ML shear **(C)** between initial (blue) and terminal double limb stance (red), respectively. Circles and error bars indicate the mean value and the standard deviation in each DLS, respectively. # and ## represent significant differences between the current and previous speeds at *p* < .05 and *p* < .01, respectively. $ and $$ represent significant differences between initial and terminal double limb stance at *p* < .05 and *p* < .01, respectively. **(D–F)**: comparisons of peak vGRF timing asymmetry **(D)**, AP shear asymmetry **(E)**, and ML GRF shear asymmetry **(F)** between individuals with uTFA (purple) and the control group (gray). Circles and error bars indicate the mean value and the standard deviation in each group, respectively. * and ** represent significant differences in asymmetry ratio between the individuals with UTFA and control groups at *p* < .05 and .01, respectively.

For peak vGRF timing (% DLS of peak vGRF timing), there was no significant difference observed between the initial and terminal DLS at all walking speeds (Figure 5A). For the initial DLS, there was a significant difference between current and previous speeds at walking speeds between 3.0 km/h and 4.0 km/h (*p* < .01 or *p* < .05). For the terminal DLS, there was a significant difference between current and previous speeds at 5.0 km/h or higher (*p* < .01 or *p* < .05).

For AP shear (% DLS of AP shear) in individuals with uTFA, there was a significantly higher % DLS in terminal DLS AP shear than the initial DLS at all walking speeds tested (*p* < .01) (Figure 5B). There were also no significant differences observed between adjacent walking speeds in AP shear % DLS in both the initial and terminal DLS at all tested walking speeds.

For ML shear (% DLS of ML shear) in individuals with uTFA, there were significant differences between the initial and terminal DLS (*p* < .01 or *p* < .05) (Figure 5C). Only at 2.5 km/h and 3.0 km/h for the initial DLS was there a significant difference between current and previous walking speeds (*p* < .05).

There were only significant differences found in the peak vGRF asymmetry ratio between individuals with uTFA and the control group at 2.0 km/h (*p* < .05) (Figure 5D). There were also no significant differences observed between adjacent walking speeds in peak vGRF asymmetry ratio at all tested walking speeds for both groups.

For the AP shear asymmetry ratio, there was a significant difference at all tested walking speeds between individuals with uTFA and the control group (*p* < .01) (Figure 5E). Only at 4.0 km/h for individuals with uTFA was there a significant difference between current and previous walking speeds for the AP shear asymmetry ratio (*p* < .01).

There was a significant difference in ML shear asymmetry ratio between individuals with uTFA and the control group at all tested walking speeds (*p* < .01 or *p* < .05) (Figure 5F). For the control group, there was no significant difference between adjacent speeds in ML shear asymmetry ratio at all tested speeds. For individuals with uTFA, there was only a significant difference at 2.5 km/h between current and previous speeds (*p* < .05).

## 4 Discussion

The first aim of this study was to compare GRFs between the initial and terminal DLS in individuals with uTFA. Our results showed that while the vertical and AP components of GRFs significantly differed between the initial and terminal DLS of individuals with uTFA, the ML components of GRF displayed no significant differences (Figure 4). This partially agreed with our study’s first hypothesis that the vertical, AP, and ML components of GRF between the initial and terminal DLS would differ in individuals with uTFA because of the switching of the leading limb between the intact and prosthetic limb. The second aim of the study was to compare the asymmetry ratio of GRFs between individuals with uTFA and those without amputation. Our results showed significant differences in the asymmetry ratios of peak vGRF and AP GRF but not ML GRF for all tested speeds between individuals with uTFA and those without amputation (Figure 4). This partially agreed with our study’s second hypothesis that the magnitude of asymmetry ratio of GRFs would be greater in uTFAs when compared to the able-bodied controls.

The peak vGRF during the initial DLS was significantly higher than the terminal DLS for all tested walking speeds in individuals with uTFA (Figure 4A). This result could be explained as the individuals with uTFA rely more heavily on the intact limb during initial DLS than the terminal DLS where the prosthetic limb is leading. The peak vGRF asymmetry ratio (Figure 4D) also supports this explanation since asymmetry ratios in the individuals with uTFA were consistently just below 1.0, whereas in the control group it was consistently around 1.0 indicating nearly perfect symmetry and justified with prior studies on able-bodied individuals (Polk et al., 2016). This indicates individuals with uTFA generate more vGRF when the intact limb is leading during the initial DLS to prepare for the subsequent single limb stance on the intact limb, compared to the terminal DLS. Perhaps due to pain and discomfort on the residual limb or other reasons, they prefer to not rely on the prosthetic limb as much when preparing for the subsequent single limb stance on the prosthetic limb. Even during standing, individuals with amputation load less than 40% of their body weight onto the prosthetic limb (Vrieling et al., 2008). This is augmented by our results that found the asymmetry of vGRF begins even while the other limb is still in contact with the ground. Previous findings of vGRF asymmetries between the intact and prosthetic limb in individuals with uTFA are also in agreement (Castro et al., 2014; Soares et al., 2016; Rutkowska-Kucharska et al., 2018).

Terminal DLS generated larger propulsion forces when compared to initial DLS during all walking speeds (Figure 4B). The findings agreed with previous studies on individuals with unilateral transtibial amputation which found reduced push-off power in the prosthetic limb when compared to the intact limb (Morgenroth et al., 2011; Adamczyk and Kuo, 2015). This result could be interpreted that individuals with uTFA favor their intact leg as the intact limb is the trailing limb during terminal DLS which generates propulsive force to push-off and move the individual in the forward direction. This could be an effect of the different spatiotemporal strategies that individuals with amputation use between the initial and terminal DLS to coordinate the propulsive forces necessary to control advancement velocity during walking (Michel and Chong, 2004). This is likely to contribute to the asymmetrical nature of gait commonly displayed in individuals with amputation. The AP GRF asymmetry ratio of the control group stayed consistently close to 1.0, indicating that able-bodied individuals do not favor either leg to generate propulsive forces while walking, agreeing with a prior study (Polk et al., 2016). In contrast, the AP GRF asymmetry ratio for individuals with uTFA shows that they favor their intact leg at least twice as much when generating propulsive forces (Figure 4E).

There were no significant differences between ML GRF measured during the terminal and initial DLS for individuals with uTFA (Figure 4C). However, the ML GRF asymmetry suggests that even the control group cannot produce symmetrical ML GRF with increasing speeds (Figure 4F). This agreed with a previous study that found significant asymmetries in mediolateral forces and impulses during walking in able-bodied individuals (Polk et al., 2016). Another study suggested that gravity and the abductor muscles contribute to the larger medial GRF required for faster walking, and proposed that individuals with weaker abductor muscles have trouble generating the larger medial GRF necessary for faster walking (John et al., 2012). Perhaps the ML GRF asymmetry results found in our study could be attributed to differences in individual ability to cope with faster walking speed, which may be explained by differing degrees of imbalances in the abductor muscles of the control group participants. Another explanation could be due to the stepping strategy of both the control group and individuals with amputation. For individuals with amputation, a previous study has found inconsistencies in the placement of the prosthetic limb, due to the lack of active lateral ankle movement resulting in a wider step width for stability (Hof et al., 2007). The treadmill used for this study also had limited dimensions, with an especially small margin between the feet and treadmill border surfaces. This could have restricted the gait of the participants, particularly for the ML plane. These could be reasons why both the control group and individuals with uTFA showed ML GRF asymmetry but were not statistically different from each other.

The duration spent in terminal DLS was longer than in initial DLS for individuals with uTFA. However, although the average duration spent in terminal DLS was longer than in initial DLS for all walking speeds in individuals with uTFA, there were only statistically significant differences at the slower walking speeds of 2.0 km/h or 2.5 km/h (*p* < .05) (Figure 3A). At faster walking speeds, our findings indicate that the time spent in initial and terminal DLS gets closer together and was no longer statistically significant. Our findings during slower walking speeds agreed with previous studies on the duration of DLS where individuals with uTFA took longer periods to transition into the single limb stance on the prosthetic limb during the terminal DLS (Isakov et al., 1997; Amma et al., 2021). During the initial DLS in individuals with uTFA, the more reliable intact limb is leading, allowing for a smooth transition of CoM to the subsequent single limb stance on the intact limb. In contrast, during the terminal DLS the prosthetic limb is leading, and perhaps individuals with uTFA require more time to shift their CoM to the prosthetic limb to prepare for the swing phase. Additionally, slower walking speeds might increase the instability in gait for individuals with uTFA, contributing to the longer duration of DLS when the prosthetic limb is leading. When comparing the asymmetry ratio of duration between the terminal and initial limb, there were significant differences between individuals with uTFA and those without amputation at walking speeds of 4.0 km/h or less (Figure 3B). The asymmetry ratio for the control group stayed very close to 1.0 indicating nearly perfect symmetry in duration between the terminal and initial DLS, while for individuals with uTFA, at slower walking speeds, there was a greater difference between the terminal and initial DLS (Figure 3B). Perhaps at faster walking speeds, the asymmetries in duration spent in the terminal and initial DLS could be minimized for individuals with uTFA, due to the necessity of a quickened terminal DLS to cope with the faster speed. Moreover, self-stabilizing dynamics might simplify the control of locomotion, which may decrease the reliance on the intact limb for stability and balance (Geyer et al., 2006).

Regarding the effect of walking speed on temporal parameters during DLS, there was no discernable pattern in the % of DLS for peak vGRF timing (Figure 5A). It appears that even for the control group, there were varying peak vGRF timing asymmetries across walking speeds (Figure 5D). However, the AP and ML shear temporal parameters could provide some insight into the gait strategies of individuals with uTFA. For individuals with uTFA, the % DLS where anteroposterior force transitioned was relatively consistent for the initial DLS across all speeds (Figure 5B). However, for the terminal DLS, the % DLS decreased with increasing speed. This could indicate that with increasing speeds individuals with uTFA need to brake earlier on the prosthetic limb because they cannot effectively generate braking force when compared to the intact limb. There are also clear differences in the AP shear asymmetry values between the control group, which was close to perfect symmetry across all speeds when compared to individuals with uTFA, further contributing to the idea that the prosthetic limb cannot generate braking force effectively when compared to an intact or able-bodied limb (Figure 5E).

Additionally, for ML shear, individuals with amputation had a relatively consistent % DLS when the transition occurred for the terminal DLS, whereas the initial DLS increased with increasing walking speed (Figure 5C). Perhaps, this could be interpreted that individuals with uTFA favor leaving their CoM on their intact limb, transitioning at a later % DLS with increasing walking speed to improve their stability. This thought could be supported by the preference even while standing still for individuals with amputation to load more of their body weight on their intact limb (Vrieling et al., 2008), as well as a previous study that reported a larger range of variation in angular momentum in the AP or ML direction differentiating the instability of gait in individuals with uTFA and able-bodied individuals (Al Abiad et al., 2020). The control group’s ML shear (% DLS) was also becoming more asymmetrical as walking speed increased, similar to individuals with uTFA (Figure 5F). This is similar to the effect of walking speed on ML GRF, indicating that both individuals with uTFA and without amputation become more unstable mediolaterally with increasing walking speed. This agreed with prior studies on able-bodied individuals finding asymmetries in ML parameters caused by muscle recruitment differences (John et al., 2012). The AP shear asymmetrical ratios agree with previous findings that individuals with amputation have lower stability than able-bodied subjects (Paradisi et al., 2019).

Individuals with uTFA appear to have apparent vertical or AP GRF asymmetries across increasing walking speeds (Figures 4D, E). Knee osteoarthritis is more prevalent in individuals with amputation (Struyf et al., 2009; Russell Esposito and Wilken, 2014), and due to their asymmetrical nature of gait, higher GRFs experienced by the intact limb are likely to be a possible cause (Silverman and Neptune, 2014). Gait asymmetry correlates with significant increases in the load borne by the intact limb (Castro et al., 2014), and although our results indicated that increased walking speed resulted in increased GRFs overall during DLS, the asymmetrical ratio did not necessarily change. Therefore, clinical applications could be that while advising individuals with amputation to walk at slower speeds may lower GRFs in the intact limb, it may not reduce the asymmetrical nature of their gait. This is important because greater loading on the intact limb may lead to knee osteoarthritis or musculoskeletal degenerative disorders (Norvell et al., 2005; Kaufman et al., 2011; Devan et al., 2014) although the direct association between GRFs (amount of loading and asymmetry) and these disorders during DLS has not been clarified yet and requires further investigations.

Several limitations should be considered during the interpretation of our results. First, our participants walked on an instrumented treadmill to collect data on the parameters analyzed while controlling the walking speed. Some studies have found differences when collecting data from individuals with amputation for parameters such as variability of gait asymmetry and higher metabolic costs when using instrumented treadmills compared to overground (Traballesi et al., 2008; Starholm et al., 2010; Kluitenberg et al., 2012). Second, we had 15 participants in each group (Table 1), which limits the statistical power of our study. However, the low sample size is common in studies of individuals with amputation as it is difficult to recruit participants that can meet the criteria of the study (Burkett et al., 2003; Mengelkoch et al., 2017). Third, whether the amputated leg of the individuals with uTFA was their dominant or non-dominant leg was not recorded, and neither was there any documentation of the dominant leg in the control group. Prior studies have reported limb dominance to be a factor in functional asymmetries, particularly in ML variations during walking (Sadeghi et al., 2000; Polk et al., 2016) and possibly in AP parameters in relationship with increasing walking speed (Rice and Seeley, 2010). Fourth, different types of prosthetic knees and feet were used by individuals with uTFA in the study. While mechanical knee joints (hydraulic or pneumatic) and energy storage and return feet were generally used by the participants, variations in components and designs could also change gait symmetry (Segal et al., 2006; Kaufman et al., 2012) or vary GRF loading responses (Gard and Konz, 2003). For instance, some prosthetic feet are designed with a split-keel blade, such as the Highlander and Dyna Trek feet, which could provide better ML stability affecting ML forces. Therefore, care should be taken before generalizing our results to the general population of individuals with amputation. Finally, only GRF and temporal parameters were collected in this study. Data on CoM movement or lower-limb joint kinematics during DLS would provide information on the relationship between GRF asymmetries and motion during gait. Therefore, a future study should consider kinematics, kinetics and CoM movement comprehensively to analyze gait of individuals with uTFA.

In conclusion, this study found that there are significant differences in the vertical and AP components of GRF during DLS of gait in individuals with uTFA depending on whether the intact (initial DLS) or prosthetic (terminal DLS) limb is leading. There are also significant differences in the asymmetry ratios of peak vGRFs and AP GRFs between individuals with uTFA and able-bodied individuals. However, for ML GRFs there were no statistically significant differences between the initial and terminal DLS of individuals with uTFA, as well as their asymmetry ratios when compared to able-bodied individuals. These findings demonstrated the asymmetrical nature of gait in individuals with uTFA even when both limbs are in contact with the ground during DLS. Increasing walking speed reduced asymmetry of some temporal parameters in individuals with uTFA, but increased GRF loading asymmetries. Therefore, individuals with uTFA have further asymmetrical loading between the two DLS with increasing walking speed but reduced asymmetrical temporal parameters due to the necessary gait adjustments with increasing walking speed. Therefore, increased walking speed in individuals with uTFA may further escalate the susceptibility to knee osteoarthritis or musculoskeletal disorders. This study also suggested that individuals with uTFA would utilize different adaptations and compensation strategies at initial and terminal DLS during gait. Further study is needed to investigate and develop optimal gait adaptation and compensation strategies for individuals with uTFA to maximize stability of gait while minimizing gait asymmetry and loading on the intact limb.

## Data Availability

The raw data supporting the conclusion of this article will be made available by the authors, without undue reservation.

## Abbreviations

ANOVA, Analysis of variance; AP, Anteroposterior; BW, Body weight; CoM, Center of mass; DLS, Double limb stance; Fx, Mediolateral; Fy, Anteroposterior; Fz, Vertical; GRF, Ground reaction force; IC, Initial contact; ML, Mediolateral; uTFA, Unilateral transfemoral amputation; vGRF, Vertical ground reaction force.
